# Subsecond Sensory Modulation of Serotonin Levels in a Primary Sensory Area and Its Relation to Ongoing Communication Behavior in a Weakly Electric Fish

**DOI:** 10.1523/ENEURO.0115-16.2016

**Published:** 2016-11-02

**Authors:** Haleh Fotowat, Erik Harvey-Girard, Joseph F. Cheer, Rüdiger Krahe, Leonard Maler

**Affiliations:** 1 Department of Cellular and Molecular Medicine, University of Ottawa, Ottawa, Ontario K1H 8M5, Canada; 2 Department of Anatomy and Neurobiology, Department of Psychiatry, University of Maryland, Baltimore, Maryland 21201; 3 Department of Biology, McGill University, Montréal, Québec H3A 1B1, Canada

**Keywords:** 5-HT, communication behavior, electrosensory, fast-scan cyclic voltammetry, serotonin, weakly electric fish

## Abstract

Serotonergic neurons of the raphe nuclei of vertebrates project to most regions of the brain and are known to significantly affect sensory processing. The subsecond dynamics of sensory modulation of serotonin levels and its relation to behavior, however, remain unknown. We used fast-scan cyclic voltammetry to measure serotonin release in the electrosensory system of weakly electric fish, *Apteronotus leptorhynchus*. These fish use an electric organ to generate a quasi-sinusoidal electric field for communicating with conspecifics. In response to conspecific signals, they frequently produce signal modulations called chirps. We measured changes in serotonin concentration in the hindbrain electrosensory lobe (ELL) with a resolution of 0.1 s concurrently with chirping behavior evoked by mimics of conspecific electric signals. We show that serotonin release can occur phase locked to stimulus onset as well as spontaneously in the ELL region responsible for processing these signals. Intense auditory stimuli, on the other hand, do not modulate serotonin levels in this region, suggesting modality specificity. We found no significant correlation between serotonin release and chirp production on a trial-by-trial basis. However, on average, in the trials where the fish chirped, there was a reduction in serotonin release in response to stimuli mimicking similar-sized same-sex conspecifics. We hypothesize that the serotonergic system is part of an intricate sensory–motor loop: serotonin release in a sensory area is triggered by sensory input, giving rise to motor output, which can in turn affect serotonin release at the timescale of the ongoing sensory experience and in a context-dependent manner.

## Significance Statement

Serotonin is a key modulator of neural activity throughout the brains of all vertebrates. Understanding the function of serotonin in sensory processing is critical as its disruption in sensory perception is an important element of many neurological disorders. We studied the temporal dynamics of serotonin release in response to communication signals in weakly electric fish. We found that serotonin release is temporally tightly linked to communication stimuli and likely depends on an individual’s past experience. Interestingly, the communication behavior of a fish can also affect the time course of serotonin release. Our results are the first to shed light on the subsecond dynamics of serotonin in a sensory area and its relation to ongoing behavior.

## Introduction

The serotonergic [5-hydroxytryptamine (5-HT)] system affects targets throughout the CNS and is highly conserved across vertebrates (Jacobs and Azmitia, 1992). Serotonergic innervation of the CNS arises mainly from medial and dorsal raphe nuclei (Berger et al., 2009). Altered levels of 5-HT contribute to mental disorders like depression, schizophrenia, and autism (Müller and Jacobs, 2010). In the context of agonistic encounters, lower 5-HT levels have been associated with increased aggression, although some studies underline the dependence of this effect on the history of the animal, the behavioral context, and the presence of differential changes across brain regions (Summers et al., 2005; Nelson and Trainor, 2007; Silva et al., 2013).

5-HT significantly affects the response properties of sensory neurons across vertebrates (Hurley et al., 2004). The effect of 5-HT on sensory responses has been largely studied through electrical stimulation of serotonergic fibers, through microinjection of its receptor agonists or antagonists (Rogawski and Aghajanian, 1980; Waterhouse et al., 1990; Hurley and Pollak, 2005; Dacks et al., 2009; Petzold et al., 2009), and through their optogenetic excitation (Dugué et al., 2014). Electrochemical techniques are particularly valuable in unraveling the release dynamics of endogenous monoamines in the brain (Robinson et al., 2008). Sensory modulation of 5-HT levels in the inferior colliculus (IC) of mice has been studied using differential pulse voltammetry, shedding light on the temporal dynamics of 5-HT release on a timescale of minutes (Hall et al., 2010). Serotonin release evoked by tail-nerve shocks was measured in the dissected nervous system of *Aplysia* at the subsecond timescale using chronoamperometry, elucidating its role in learning and memory, particularly through the spatially specific release pattern (Marinesco and Carew, 2002; Marinesco et al., 2006). Sensory modulation of 5-HT dynamics at the subsecond timescale in relation to ongoing natural behaviors, however, has not yet been characterized.

We used South American gymnotiform weakly electric fish, *Apteronotus leptorhynchus*, to measure 5-HT release in the first processing stage of electrosensory input, the electrosensory lobe (ELL) of the hindbrain *in vivo*. These fish generate a quasi-sinusoidal electric field through discharges of an electric organ (EODs; Krahe and Maler, 2014). The EOD frequency (EODf) is specific to the individual, with males having higher frequencies than females (Zakon et al., 2002). The EOD of conspecifics can be mimicked and presented to a fish in an experimental tank through the application of a weak sinusoidal voltage to a pair of electrodes placed at a distance from the fish, such that the resulting electric field casts a global image on the body of the fish (Clarke et al., 2015). Such mimicking of conspecific signals triggers behavioral responses that are indistinguishable from those observed during interaction with real conspecifics and have been widely used to study electrocommunication behavior. For example, the characterization of the jamming avoidance response was entirely based on such electrosensory mimic stimuli (Heiligenberg 1991). During interaction with a conspecific, the electric fields summate and generate amplitude modulations (AMs) at the difference frequency (Df) of the individual EOD frequencies (Heiligenberg, 1991). The AM frequency provides information about the sex of the conspecific (e.g., same-sex and opposite-sex interactions result in small and large Dfs, respectively; Walz et al., 2013). Tuberous electroreceptor afferents encode EOD AMs and provide somatotopic input to three segments of the ELL, each specialized in processing distinct electrosensory stimuli (Esense-stim): lateral segment (LS), centrolateral segment, and centromedial segment (CMS; Krahe and Maler, 2014). The LS specifically receives strong serotonergic input from raphe nuclei (Johnston et al., 1990; Deemyad et al., 2011), is specialized in processing communication signals called chirps (Marsat et al., 2012), and is necessary for chirp production (Metzner and Juranek, 1997). Chirps are brief increases in EOD frequency, most commonly produced by males during agonistic encounters (Zakon et al., 2002). Chirping behavior is under the influence of 5-HT: ventricular injection of 5-HT (Maler and Ellis, 1987) and agonistic compounds of 5HT_2_ receptors alike (Smith and Combs, 2008) reduce chirping. Local injection of 5-HT in LS and stimulation of raphe nuclei also reduce chirping (Deemyad et al., 2013). Serotonin injection increases burst firing and decreases first spike latency in LS pyramidal cells, rendering them more sensitive to conspecific chirps (Deemyad et al., 2013). This effect is exerted through the inhibition of small-conductance calcium and M-type potassium currents (Deemyad et al., 2011), mediated by 5-HT_2_-like receptors (Larson et al., 2014). The pattern of 5-HT release in LS, and its relation with chirp production, however, remains elusive. In this study, we used fast-scan cyclic voltammetry (FSCV; Dankoski and Wightman, 2013) to detect electrosensory modulation of 5-HT release in the LS, and characterized its relation to chirping behavior.

## Materials and Methods

All animal procedures were performed in accordance with the regulations of the animal care committee of the University of Ottawa. *Apteronotus leptorhynchus* of either sex were used for all experiments.

### Carbon fiber microelectrode fabrication

Seven-micrometer-diameter carbon fibers (Goodfellow Cambridge) were used to construct carbon fiber microelectrodes (CFMEs). The carbon fiber was drawn into a glass capillary (outer diameter, 1.2 mm; inner diameter, 0.68 mm; A-M Systems) by suction, and the ensemble was pulled using a horizontal puller (P1000; Sutter Instruments). The carbon fiber extending through the tip of the pulled pipette was cut and trimmed to a length of 120–140 µm. A thin copper wire was inserted from the other end of the capillary and connected to the carbon fiber using silver print (Silver Print II, GC Electronics). The other end of the wire was soldered to a gold pin used to connect the electrode to the voltammetry head stage, and was secured to the glass capillary using heat shrink tubing. The CFME tip was electroplated with 5% Nafion [LIQUion (LQ-1105-MeOH), Ion Power] to increase sensitivity to 5-HT (Hashemi et al., 2009). The CFME tip was placed in Nafion solution and +1 V constant voltage was applied to it relative to an Ag/AgCl electrode for 30 s. The CFME was next air dried for 10 s and then placed in a convection oven at 70°C for 10 min. The electrodes were stored dry in a container before being used.

### FSCV

**Figure 1.**
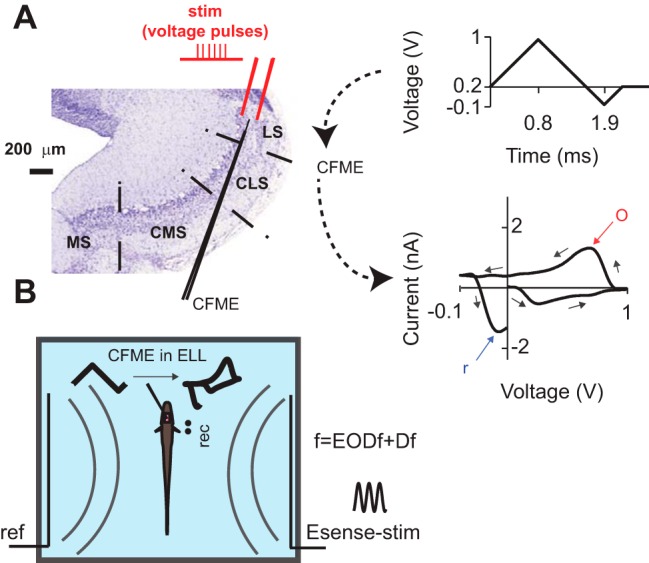
Nissl-stained transverse section of ELL, and the schematics of *in vitro* and *in vivo* experimental setups. ***A***, The stimulating bipolar electrode and the CFME were placed in the ELL pyramidal cell layer. A triangular waveform (top right) sweeping between −0.1 and +1 V at 1000 V/s was applied to the CFME once every 100 ms. The voltage was kept at 0.2 V between the scans. The background-subtracted CV (bottom right, a representative CV from slice recordings) was calculated for each scan. o, Oxidation peak; *r*, reduction peak. Arrows point to the temporal order of the points in the CV. ***B***, Sketch of the *in vivo* setup: Esense-stim was presented to the fish with a carrier frequency (f) equal to the EODf of the fish plus Df. ref, Reference electrode; rec, EOD-recording electrode. Curved gray lines schematize the existence of a global electric field but do not represent the actual, more intricate orientation of the electric field lines.

The voltammetry headstage was manufactured by Electronics and Materials Engineering (EME) and was used to apply a triangular waveform to the CFME relative to an Ag/AgCl reference electrode and to record the resulting current. Tar-Heel CV software (University of North Carolina, Chapel Hill, NC) was used together with data acquisition boards (NI-PCI-6052E and NI PCI-6711, National Instruments) for generating the triangular waveform and acquiring the resulting current. The waveform had an N shape (Fig. 1*A*, right), swept between −0.1 and 1 V, at 1000 V/s, and rested at 0.2 V in between the scans (Jackson et al., 1995). In all experiments (*in vitro* and *in vivo*), prior to the start of recording, the triangular waveform was applied to the CFME at 60 Hz for 20 min to overoxidize the carbon fiber, and thereby increase its surface area and sensitivity (Robinson et al., 2008). Next, the same waveform was applied to the electrode at the recording frequency (10 Hz) for 10 min to achieve a stable background (non-faradic) current at this frequency. The average background cyclic voltammogram (CV) was calculated in a 1 s window (10 scans) immediately prior to stimulus onset and subtracted from all subsequent CVs to facilitate the detection of smaller faradic currents resulting from redox reactions of 5-HT (Robinson et al., 2008).

Color plots depict time on the *x*-axis, the voltage of the applied triangular waveform on the *y*-axis, and the current amplitude in false color.

### *In vitro* FSCV recordings and electrical stimulation

*A. leptorhynchus* were anesthetized with tricaine methanesulfonate (MS-222; Aqua Life, Syndel Laboratories) in oxygenated deionized water. The ELL was removed, and transverse slices of 350 μm thickness were prepared on a vibratome, while the ELL was immersed in ice-cold artificial CSF (ACSF) containing the following (in mm): 135 NaCl, 2 KCl, 1.25 KH_2_PO_4_, 1.5 CaCl_2_, 1.5 MgSO_4_, 24 NaHCO_3_, 10 d-glucose, and 5 Na-ascorbate. Slices were maintained in an interface chamber in ACSF at room temperature for 30 min before recording. Gold-plated bipolar tungsten electrodes (interpole distance, 150 μm; FHC) were used to deliver 100 μs pulses at 60 Hz to the pyramidal cell layer (PCL; Fig. 1*A*). This stimulation frequency was previously reported to be efficient for stimulating dorsal raphe neurons *in vitro* (Bunin et al., 1998). The interstimulus interval was 5–10 min. The CFME was inserted into the slice (depth, ∼150 μm) at a shallow angle (∼30^°^) and placed at equal distance (∼350 μm) from either pole of the stimulating electrode (Fig. 1*A*). The 5-HT reuptake inhibitor citalopram hydrobromide (Sigma-Aldrich) was used to confirm the identity of the measured analyte. To compare the stimulation-evoked responses before and after citalopram application, the currents corresponding to control and citalopram trials were normalized to the average peak amplitude of control trials for each fish.

### *In vivo* FSCV recordings, electrosensory and auditory stimuli

*A. leptorhynchus* were deeply anesthetized with MS-222, and their ELL was exposed by craniotomy. They were then locally anesthetized at the wound margin by application of 20% benzocaine cream (Orajel, Church & Dwight), and allowed to recover from general anesthesia, immobilized using curare [(+)-tubocurarine chloride, Sigma-Aldrich], transferred to the experimental tank, and respirated with a continuous flow of aerated water through a tube inserted into the mouth of the fish. This protocol does not appear to result in stress or pain (Hitschfeld et al., 2009) and has, therefore, been accepted by the animal care committee of the University of Ottawa, and is also in accord with Canadian and Society for Neuroscience guidelines on animal care. An Ag/AgCl ground electrode was inserted into the neck muscle and connected to the system ground to reduce the contamination of the recording by the EOD of the fish. The CFME was first connected to an extracellular amplifier and lowered toward LS (or CMS in control experiments). The reference Ag/AgCl electrode was placed dorsal from the CFME recording electrode on the surface of the brain. We verified the location of the electrode tip in the PCL based on the known depth of the PCL (∼500 μm), and the increase in the level of population spiking activity was measured extracellularly by the CFME upon its arrival at the LS-PCL. The fish were allowed to acclimate to the experimental setup for at least 1 h before the start of FSCV recording. Redox currents evoked in response to 20-s-long Esense-stim simulating the presence of conspecifics (Krahe and Maler, 2014; Clarke et al., 2015) and loud auditory stimuli (Aud-stim) were measured. Aud-stim consisted of intense pure tones with frequencies between 700 and 900 Hz played on a pair of speakers placed near the tank. Esense-stim were generated by applying a weak sinusoidal voltage waveform to the tank water through a pair of carbon electrodes (diameter, 8.3 mm) placed on either side of the tank (Fig. 1*B*), creating a global electric field in the tank water (Heiligenberg, 1991; Clarke et al., 2015). The frequency of the field was chosen to be in the range of naturally occurring EOD frequencies of *A. leptorhynchus* as follows: −100, −80, −60, −40, −20, −10, −5, 5, 10, 20, 40, 60, 80, and 100 Hz. The frequency of the Esense-stim was controlled on-line such that it had a constant Df relative to the EODf of the fish. The EOD of the fish was recorded using another pair of carbon electrodes placed near the animal (Fig. 1*B*, rec). The amplitude of the Esense-stim was calibrated such that the resulting AMs measured next to the skin of the fish were between 25% and 50% of the EOD of the fish amplitude. This value corresponds to the range of AMs the fish experiences during agonistic interactions and chirp production (Hupé and Lewis, 2008; Fotowat et al., 2013). Each trial consisted of 60 s of voltammetric measurements. The interval between stimulation trials was 5–10 min. Each stimulation trial was followed by a “blank” trial in which the recording was performed in the absence of Esense-stim or Aud-stim.

### Data analysis

In our recordings, the average peak oxidation current was 1.22 nA (SD, 0.95 nA; *n*_trials_ = 114; *n*_fish_ = 9). In this range, the current amplitude changes linearly with concentration (Dankoski and Wightman, 2013). For a typical electrode used in our recordings, each nanoampere of current corresponded to an ∼80 nm change in 5-HT concentration. For the current study, all population analysis for *in vivo* experiments was performed on currents normalized to their maxima for each fish. Esense-stim and Aud-stim presentations were controlled using Spike2 software (Cambridge Electronic Design Limited). MATLAB (MathWorks) was used for all subsequent data analysis. The area under the curve (AUC) was calculated as the integral of the normalized current using trapezoidal numerical integration, and was used to quantify the normalized response magnitude over the stimulus duration (20 s). For blank trials, the AUC was calculated from 5 to 25 s after recording onset. Response latency was calculated as the time since stimulus onset when the current amplitude exceeded twice the average “noise” level, defined as the SD of the normalized current measured in a 4 s window prior to stimulus onset. Any trial that showed a threshold crossing at any time after stimulus onset (or 5 s after recording onset in blank trials) and up to 15 s after its offset was considered a response trial. Response probability was calculated as the ratio of response to no-response trials for either stimulation or blank trials. We calculated average normalized current amplitudes using all trials, whether or not a response was present (current passed the threshold). Response duration was calculated as the time between the response onset and the first time the current dropped below noise level. The Kruskal–Wallis test (KWT) was used to compare the medians of two groups, and Tukey–Kramer multiple comparisons were used for comparison among three or more groups. A *t* test was used to test whether the mean of a Gaussian distribution was significantly different from zero. The number of fish tested and the number of trials are denoted by *n*_fish_ and *n*_trials_, respectively. The Pearson correlation coefficient and its significance calculated using *t* statistics are denoted by ρ and *p*, respectively.

## Results

### Electrical stimulation of 5-HT fibers evokes anatomically specific 5-HT release *in vitro*

**Figure 2.**
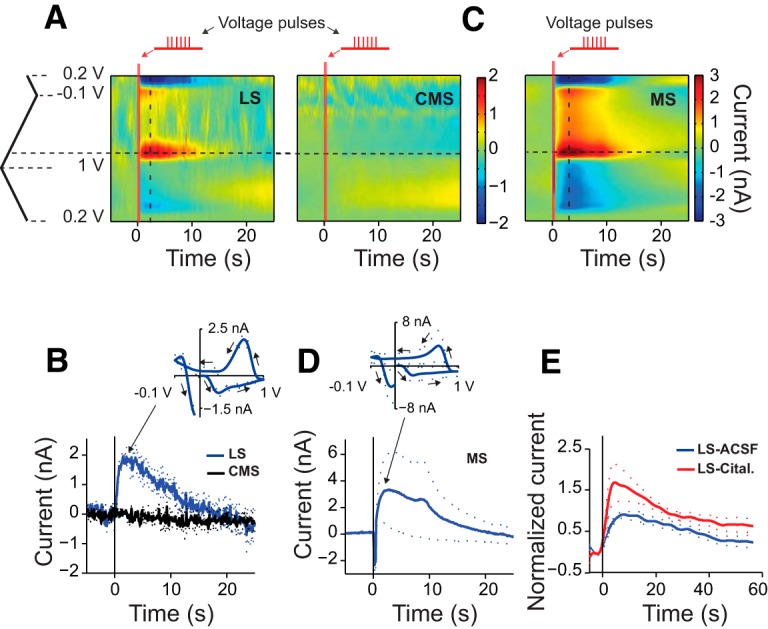
*In vitro* electrical stimulation in ELL-PCL evokes 5-HT release in the LS and MS, but not CMS. ***A***, Representative average color plot for electrical stimulation trials in LS (left panel, *n*_trials_ = 3, *n*_fish_ = 1) and CMS (right panel, *n*_trials_ = 3, same fish). Each trial for electrical stimulation consisted of the application of 24, 0.1-ms-wide, 20 V voltage pulses at 60 Hz. Vertical red bars depict the onset and duration of electrical stimulation. Horizontal and vertical dashed lines correspond to the peak oxidation voltage and time of the peak current, respectively. ***B***, Time-course of the average (±SEM) current at the peak oxidation voltage for stimulation and recording in LS (blue) and CMS (black). Same fish as in ***A***, *n*_trials_ = 3 for each region. Oxidation peak 0.76 V (SD = 0.01 V), reduction peak 0.17 V (SD = 0.01 V). Inset depicts the average (±SEM) CV for LS measured at peak current for these trials. ***C***, Representative average color plot for electrical stimulation trials in MS (*n*_trials_ = 3, *n*_fish_ = 1). Each trial for electrical stimulation consisted of the application of 24, 0.1-ms-wide, 60 V voltage pulses at 60 Hz. ***D***, Time-course of the average (±SEM) current at the peak oxidation voltage for stimulation and recording in MS. The locations of the redox peaks were similar to those found in LS [oxidation peak, 0.77 V (SD, 0.02 V); reduction peak, 0.14 V (SD, 0.01 V)]. Inset depicts the average (±SEM) CV. ***E***, Average normalized current measured at peak oxidation voltage before (ACSF control, black) and after incubation in citalopram (Cital., red; *n*_fish_ = 3, *n*_trials_ = 3 per condition). All currents were normalized to the average peak amplitude under control conditions for each fish. Dashed lines show the SEM.

Based on immunohistochemical studies, ELL-LS is devoid of dopamine (DA) and norepinephrine (NE; Sas et al., 1990), and 5-HT immunoreactivity is present mainly in the PCL of LS (LS-PCL) and the medial segment (MS) of the ELL (Deemyad et al., 2011). In order to verify that we could measure 5-HT concentration changes using FSCV in the brain of *A. leptorhynchus*, we recorded redox currents evoked by electrical stimulation of 5-HT fibers in slices of ELL (Fig. 1*A*; see Materials and Methods). Stimulation of LS consistently evoked redox currents at the nearby CFME (*n*_fish_ = 5; Fig. 2*A*,*B*). Stimulating CMS did not evoke such currents (*n*_fish_ = 2; Fig. 2*A*,*B*), whereas stimulating MS evoked a current in MS with similar CV as that observed in LS (*n*_fish_ = 2; Fig. 2*C*,*D*). There was, therefore, excellent correspondence between the presence of 5-HT fibers revealed by immunohistochemistry and our ability to evoke release by electrical stimulation. We found that the peak oxidation–reduction voltage was shifted relative to that obtained for exogenous 5-HT solutions with the CFME placed in the artificial CSF. It was, however, consistent with that obtained when the CFME was placed inside the slice and exogenous 5-HT was puffed on the slice surface (data not shown). Such shifts have been reported previously and are thought to be due to coating of the electrode by metabolites present in the surrounding brain tissue (Hashemi et al., 2009; Singh et al., 2011). In order to further verify that the measured redox currents were due to 5-HT release, we repeated the stimulation experiments in LS and assessed the effect of the selective 5-HT reuptake inhibitor citalopram on the stimulation-evoked current. We first measured redox currents evoked by electrical stimulation with the slice in ACSF, then incubated the same slice in the same recording chamber in 2 μm citalopram for 20 min and measured stimulation-evoked currents again (*n*_fish_ = 3; Fig. 2*E*). We found that citalopram significantly increased the peak current amplitude (*p*_KWT_ = 0.038) and the area under the curve of the stimulation-evoked current (*p*_KWT_ = 0.003). Given the known anatomical innervation patterns of serotonergic afferents in ELL, the absence of DA and NE in the ELL-LS, and the aforementioned *in vitro* control experiments, we conclude that the redox currents that we measured by FSCV in ELL-LS corresponded to 5-HT levels.

### Electrosensory stimuli modulate 5-HT levels *in vivo*

**Figure 3.**
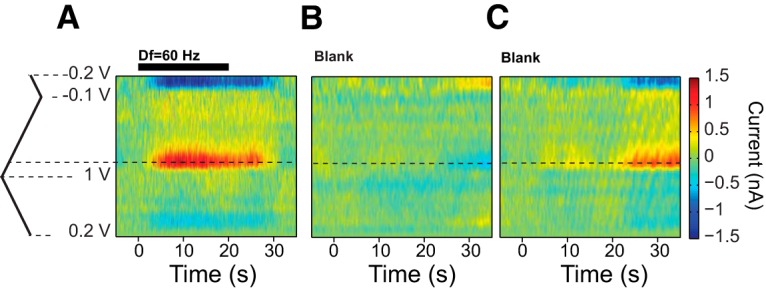
*In vivo*, 5-HT is released spontaneously as well as in response to Esense-stim. ***A***, An example color plot of the time-course of the current response elicited by the presentation of a 20-s-long Esense-stim with Df = 60 Hz. ***B***, ***C***, Example recordings in the same fish in the absence of stimulation (blank trial) without and with spontaneous release, respectively. The color scale bar on the right applies to all color plots.

We presented 20-s-long Esense-stim simulating the presence of conspecifics with Df values ranging between −100 and +100 Hz (*n*_fish_ = 9; Fig. 3*A*; see Materials and Methods). Such stimuli indeed elicited a redox current in LS (Fig. 3*A*) with a CV that resembled those obtained *in vitro* (Fig. 2*A*). Interestingly, we occasionally observed spontaneous 5-HT release events even in the absence of stimulation during blank trials (Fig. 3*B*,*C*; see Materials and Methods).

Not all Esense-stim trials, however, resulted in a change in 5-HT levels. On average, across all trials in all nine fish, the probabilities of observing evoked and spontaneous 5-HT release events were 82% (SD, 12.6%) and 51% (SD, 13%), respectively. It is important to note that these probabilities were calculated based on threshold crossing of the current anytime in a 35 s window after the stimulation onset or recording onset in the blank trials (threshold = 2× the SD of noise; see Materials and Methods). With a more stringent rule for the presence of response [e.g., threshold crossing of 3× the SD of noise, the response probability for stimulus, and blank trials will be 70% (SD, 14%) and 45% (SD, 13%), respectively]. Moreover, one could further restrict the rule for response versus no response by setting a smaller upper bound for response latency. The definition of the presence or absence of response in this sense, therefore, is sensitive to the chosen threshold and when it is crossed. To avoid this issue, we used AUC for making comparisons across different conditions, as it encompasses information about both response timing and amplitude.

**Figure 4.**
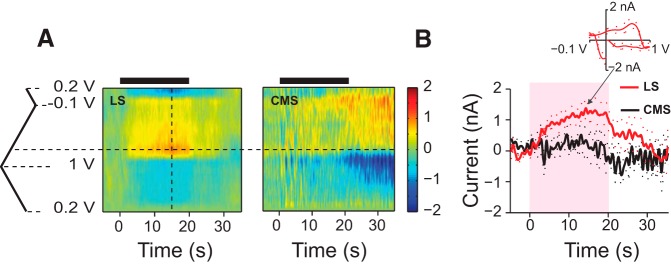
Electrosensory stimuli modulate 5-HT levels *in vivo* in LS, and not in CMS. ***A***, Representative average time-courses of redox currents in LS (left, *n*_trials_ = 7, *n*_fish_ = 1) and CMS (right, *n*_trials_ = 7, same fish) in response to Esense-stim with the same set of Df values. The recordings in CMS were acquired before moving the same electrode to LS. Horizontal and vertical dashed lines correspond to the peak oxidation voltage and time of the peak current, respectively. The thick horizontal bars on top depict the timing and duration of the stimulus. ***B***, Average current at peak oxidation voltage in LS (red) and CMS (black). Dashed lines show the SEM. Inset, Average CV at the time of the peak current in LS [red curve, oxidation peak at 0.69 V (SD, 0.06 V); reduction peak, 0.167 V (SD = 0.03)]. Time zero corresponds to stimulus onset. The pink shaded box corresponds to the duration of the stimulus.

We found large variability in the presence or absence of a 5-HT response to stimuli with different Df values in each fish. The overall response probability and AUC, however, were not significantly different for different stimulus Df values (KWT with multiple comparison, *p*_KWT_ = 0.49), and, thus, for the majority of the following analysis we pooled the data across all Df values, or within ranges of Df. Consistent with our *in vitro* experiments (Fig. 2*A*,*B*) and previous anatomical studies (Johnston et al., 1990), the Esense-stim did not evoke a response in CMS (*n*_fish_ = 3; Fig. 4).

**Figure 5.**
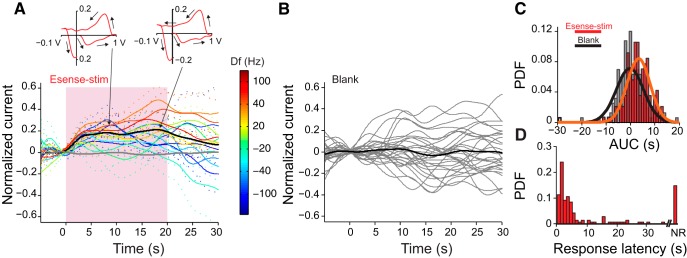
Sensory modulation of 5-HT levels in LS is phase locked to Esense-stim. ***A***, Time-course of the normalized 5-HT oxidation peak currents elicited by Esense-stim (black curve, *n*_fish_ = 9, *n*_trials_ = 142) and those that occurred spontaneously in the same group of fish (gray curve, *n*_fish_ = 9, *n*_trials_ = 158). Dashed lines show SEM. Insets on top show the CV at the two peaks indicated by the arrows. Pink shaded box corresponds to the duration of the stimulus. Colored curves show the average (dashed lines, SEM) of the current in response to stimuli with various values of Df, with the color code shown by the color bar. ***B***, Individual blank trials (gray curves) and their average (black curve) in a representative fish. Time zero corresponds to the reference time for background subtraction. ***C***, PDF plot of the AUC for Esense-stim (red filled bars) and blank trials (gray filled bars). Gaussian fits to the distributions: orange: Esense-stim [μ = 3.3 s (SE = 0.39 s), σ = 4.7 s (SE = 0.28 s)]; black: blank [μ =-0.24 s (SE = 0.44 s), σ = 5.6 s (SE = 0.32 s)]. ***D***, PDF plot for the response latency for Esense-stim trials. NR, proportion of the trials with no response. Trials used for generating ***C*** and ***D*** are the same as those depicted in ***A***.

**Figure 6.**
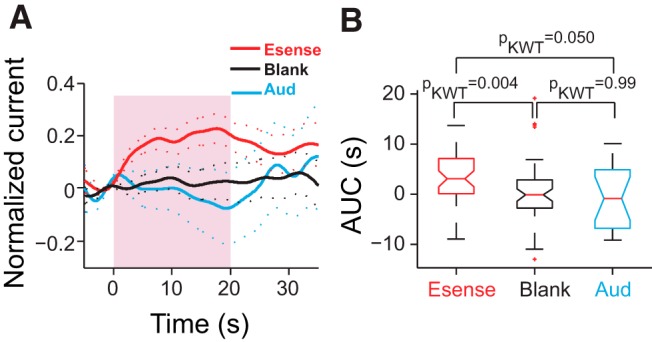
Aud-stim did not modulate 5-HT levels in LS. ***A***, Average normalized peak current (*n*_fish_ = 4) in response to Esense-stim (red, *n*_trials_ = 48) and Aud-stim (blue, *n*_trials_ = 18), and during blank trials (black, *n*_trials_ = 54). ***B***, The AUC for Aud-stim trials was not significantly different from those of blank trials and was significantly smaller than those for Esense-stim trials. The median is shown with the middle red line; the lower and upper edges of the box plots correspond to the 25th and 75th percentiles; and the whiskers mark the extent of the data points. Outliers are shown as red crosses.

Figure 5*A* shows the normalized currents averaged across all fish and all Esense-stim trials (black curve; *n*_fish_ = 9, *n*_trials_ = 142, average currents for stimuli at different Df values are additionally depicted using color codes), and all blank trials (gray curve, *n*_fish_ = 9, *n*_trials_ = 158). The timing of the spontaneous events during blank trials was not phase locked to any time point during the recording, and, therefore, the current could be increasing, decreasing, or not changing relative to the background current with equal probabilities (Fig. 5*B*; background current was set to that calculated 5 s after recording onset; see Materials and Methods). As FSCV is based on differential measurements of current relative to the background current, one can encounter negative current values (and therefore negative AUC) if a spontaneous event was occurring at the time point chosen for the background subtraction and the current amplitude decreases afterward (see Materials and Methods). Across all trials, the AUC calculated over the duration of Esense-stim was significantly larger than zero (Fig. 5*C*, red filled bars, orange Gaussian fit; mean, 3.3 s; SD, 4.7 s; *p* = 3.2 × 10^−14^; *n*_trials_ = 142, *t* test), whereas it was not significantly different from zero in blank trials (Fig. 5*C*, black open bars, black Gaussian fit; mean, −0.24 s; SD, 5.6 s; *p* = 0.60, *n*_trials_ = 158, *t* test). For Esense-stim trials, the median, and 25th and 75th percentiles for response latency were 3.5, 1.5, and 15.4 s, respectively. The peak of the probability density function (PDF) for response latencies was ∼2 s (Fig. 5*D*, red filled bars), and, for most trials (70%), the 5-HT response occurred within 7 s after stimulus onset. The time-course of the 5-HT current was variable across trials; however, the average response duration was close to that of the stimulus (mean duration, 20.6 s; SD, 11.37 s) and was significantly longer (*p*_KWT_ = 4.7 × 10^−5^) than the duration of the spontaneous current events in blank trials (mean duration blank, 14.9 s; SD, 11.5 s). A subset of the fish (*n*_fish_ = 4) was additionally presented with intense Aud-stim (see Materials and Methods) to assess the sensory modality specificity of the response. We did not observe any significant phase-locked responses to Aud-stim in LS (Fig. 6*A*). The AUC was not significantly different between blank and Aud-stim trials, but both of them were significantly smaller than the AUCs for Esense-stim trials (Fig. 6*B*).

We therefore conclude that there are modality-specific 5-HT release events in response to sensory stimuli, as well as spontaneous release events in LS. Sensory modulation of the 5-HT level is phase locked to the stimulus onset and lasts longer, with an average duration that corresponds to that of the stimulus.

### Chirp production can affect 5-HT release in LS

**Figure 7.**
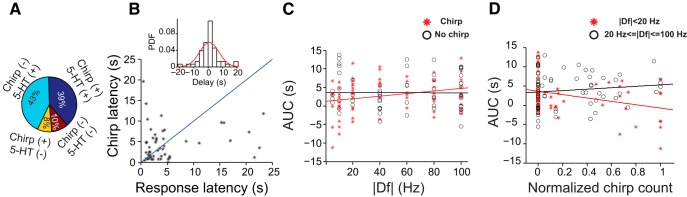
Relationship between chirp production and 5-HT response. ***A***, Pie chart depicting the proportion of trials that showed chirp and/or 5-HT responses (*n*_fish_ = 8, *n*_trials_ = 131). 5-HT release was observed equally frequently together with chirp production as without chirp production (39% vs. 43%). Trials without 5-HT release occurred equally often with chirps as without (8% vs. 10%). ***B***, Chirp latency and 5-HT response latency calculated relative to stimulus onset. The first chirp was equally likely to happen before or after 5-HT threshold crossing. The blue diagonal line depicts the unity line. Inset shows the PDF of the delays observed between 5-HT threshold crossing and the first chirp on a trial-by-trial basis (*n*_fish_ = 8, *n*_trials_ = 51). Positive delays correspond to the first chirp occurring before the 5-HT threshold crossing. The mean of the distribution was not significantly different from zero. ***C***, There was a small positive correlation between AUC and |Df| in trials in which the fish chirped (red asterisks, ρ = 0.25, *p* = 0.04, *n*_fish_ = 8, *n*_trials_ = 64), but not in those in which the fish did not chirp (black circles, ρ = −0.007, *p* = 0.95, *n*_fish_ = 8, *n*_trials_ = 67). Red line, Linear fit to the AUC of chirp trials: slope = 0.036 s^2^, intercept = 1.24 s, *R* ^2^ = 0.06). Black line, Linear fit to the AUC of no-chirp trials: slope = −0.0009 s^2^, intercept = 3.62 s, *R* ^2^ = 4.5 × 10^−5^). ***D***, We observed a small, but significant correlation between the number of chirps produced and the AUC of the 5-HT current for stimuli with a |Df| of <20 Hz (red asterisks, ρ = −0.34, *p* = 0.02, *n*_fish_ = 8, *n*_trials_ = 47), but not for stimuli with a |Df| of ≥20 Hz (black circles, ρ = 0.12, *p* = 0.27, *n*_fish_ = 7, *n*_trials_ = 84). Red line, Linear fit to the AUC of trials with a |Df| of <20 Hz: slope = −4.37 s, intercept = 3.63 s, *R* ^2^ = 0.12). Black line, Linear fit to AUC of no-chirp trials: slope = 1.87 s, intercept = 3.41 s, *R* ^2^ = 0.014).

Male fish chirped in response to stimuli mimicking the EOD of conspecifics (*n*_fish_ = 8). The mean (SD) latency of chirping onset relative to stimulus onset was 4.2 s (3.9 s; *n*_trials_ = 64). The proportion of trials where a 5-HT response was observed was similar among trials in which the fish chirped and those in which they did not chirp (Fig. 7*A*). Among trials in which both chirps and 5-HT responses were present, the average delay between the first chirp and the 5-HT response onset (i.e. the time-point of the threshold crossing for 5-HT current) was 0.86 s (SD = 6.7 s; *n*_trials_ = 51; positive delays correspond to the first chirp occurring before 5-HT response onset). Overall, in 61% of those trials the first chirp occurred prior to the 5-HT response onset (Fig. 7*B*). The mean delay, however, was not significantly different from zero, indicating that the first chirp was equally likely to occur before or after the 5-HT response onset (Fig. 7*B*, inset).

Despite a significant negative correlation between the number of chirps and the absolute value of Df (|Df|; ρ = −0.36, *p* = 0.003; see also Engler and Zupanc, 2001), there was no significant correlation between |Df| and the AUC at the individual or population level. Interestingly, however, there was a small but significant positive correlation between AUC and |Df| in the trials where the fish produced chirps (ρ = 0.25, *p* = 0.04, *n*_trials_ = 64; Fig. 7*C*, red asterisks). This correlation did not exist in the trials without chirps (ρ = −0.007, *p* = 0.95, *n*_trials_ = 67; Fig. 7*C*, black circles). Moreover, the difference in AUC between chirp and no-chirp trials was most pronounced at |Df| of <20 Hz.

Interestingly, although we did not find a significant trial-by-trial correlation between the number of chirps and AUC (ρ = −0.13, *p* = 0.12; a similar result was obtained when no-chirp trials were excluded, as follows: ρ = −0.14, *p* = 0.25), when we pooled all trials in all fish, we found a small but significant negative correlation between the number of chirps and the AUC for stimuli with a |Df| of <20 Hz (ρ = −0.34, *p* = 0.02; Fig. 7*D*, red asterisks). Such trial-by-trial correlations did not exist for stimuli with a |Df| of ≥20 (ρ = 0.12, *p* = 0.27; Fig. 7*D*, black circles). Similarly, there was no significant trial-by-trial correlation between the number of chirps and the AUC for trials in which the fish produced chirps (ρ = −0.14; *p* = 0.25).

**Figure 8.**
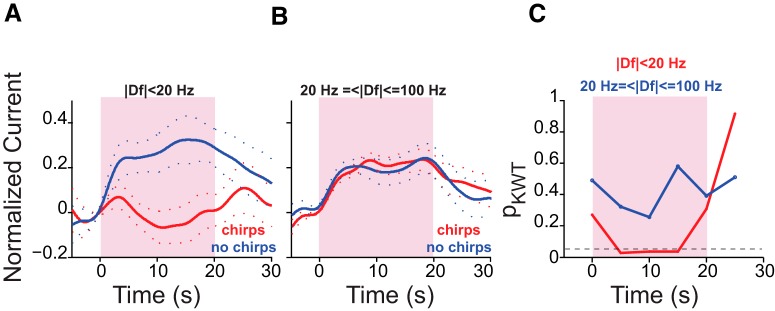
The effect of behavioral context on 5-HT response. ***A***, For trials with a |Df| of <20 Hz, the AUC of 5-HT current was significantly smaller in trials in which the fish produced chirps (*n*_fish_ = 6; chirp trials, *n*_trials_ = 14; no-chirp trials, *n*_trials_ = 14; *p*_KWT_ = 0.02). ***B***, Chirping did not affect the 5-HT response to stimuli with large |Df| values (*n*_fish_ = 4; chirp trials, *n*_trials_ = 41; no-chirp trials, *n*_trials_ = 34; *p*_KWT_ = 0.72). ***C***, KWT *p* value estimates for comparisons made between the AUC calculated over 5-s-long, nonoverlapping windows between chirp and no-chirp trials. Red, |Df| <20 Hz; blue, |Df| ≥20 Hz. The difference in AUC between chirp and no-chirp trials for |Df| values <20 Hz gains significance within the second 5 s window after stimulus onset. There was no significant difference between chirp and no-chirp trials for |Df values ≥20 Hz. The horizontal dashed line corresponds to *p*_KWT_ = 0.05.

Stimuli with small |Df| values (i.e., |Df| <20 Hz) represent the presence of similar-sized, same-sex conspecifics (context of maximal rivalry), whereas large |Df| values (|Df| ≥20 Hz) represent the presence of conspecifics of the same sex, but of different sizes (context of clear dominance or subordinance), or those of the opposite sex. We next examined the relationships among the chirping behavior of an animal, 5-HT release, and the behavioral context, as follows. We pooled all of the trials in which the fish chirped and compared the 5-HT responses to those trials where the fish did not chirp, for stimuli with small and large |Df| values (data were available for such pairwise comparisons in six fish for small |Df| values and four fish for large |Df| values). On average, the AUC of 5-HT current in response to stimuli with small |Df| values was significantly smaller in trials in which the fish chirped (Fig. 8*A*). This was not the case for stimuli with |Df| values ≥20 Hz (Fig. 8*B*). Interestingly, the difference in the time-course of 5-HT currents between the chirp/no-chirp trials gains significance in the second 5 s window after stimulus onset and loses significance after its offset (Fig. 8*C*, red curve). As the average chirp onset latency was 4.2 s, it is possible that chirp production suppresses the 5-HT response to small |Df| values, although further experiments are required to explore causality.

## Discussion

In this study, we used FSCV to measure endogenous 5-HT release in the brain of weakly electric fish in response to naturalistic electrosensory stimuli. We asked the following: does the presence of a conspecific cause 5-HT release in the LS *in vivo*, and, if so, how does that relate to evoked communication responses? Our results indicate that within seconds after the arrival of a conspecific, the 5-HT levels rise in the LS (Figs. 3*A*, 5*A*). 5-HT afferents were spontaneously active as well (Fig. 3*C*), albeit less frequently and for shorter durations. This suggests that, even in a “resting state,” there is ongoing neural activity in the brain of these fish that can modulate sensory processing. A similar conclusion was reached for motor activity and EOD discharge in related gymnotiform fish (Jun et al., 2014).

Increases in 5-HT levels occurred within a few seconds after Esense-stim onset (Fig. 5*D*) and, on average, lasted for the duration of stimulation, although we found considerable variability in response duration and probability across stimulus Df values and for different fish. As *A. leptorhynchus* are able to identify individual conspecifics based on their Df values and retain this memory for several days (Harvey-Girard et al., 2010), it is very likely that the recent encounters of the fish with other fish in their home aquaria could affect the recorded 5-HT response, and, thereby, also contribute to the response variability that we observed. Interestingly, cells in the midbrain electrosensory region torus semicircularis (TS) show selectivity for stimulus Df (Vonderschen and Chacron, 2011) and provide input to reticular formation (Carr et al., 1981). Assuming that reticular cells in weakly electric fish provide input to dorsal raphe nucleus (DRN) like they do in mammals (Ogawa et al., 2014), they would be well suited to modulate the activity of DRN neurons in a Df-specific manner.

The response latency we report here (Fig. 5*D*) is likely an overestimation of the true latency of stimulus-evoked 5-HT release in LS, as it depends on the chosen threshold value (see Materials and Methods), and is also a function of the distance of the electrode from the serotonergic terminals, over which we did not have any control. DRN neurons of cats respond to auditory and visual stimuli within 50–70 ms (Rasmussen et al., 1986). Auditory clicks evoke responses in DRN serotonergic neurons of rats with latencies as low as 20 ms (Ranade and Mainen, 2009). We expect similarly short latencies for electrosensory-evoked responses in DRN of weakly electric fish. Given the presence of direct synaptic input from DRN to the ELL-LS, a change in 5-HT level should therefore commence in this area in <100 ms after stimulus onset.

The 5-HT response was not a general “stress” response to stimulus presentation as there was no significant response to Aud-stim (Fig. 6), even though gymnotiform fish are known to be very sensitive to sound (Harvey-Girard et al., 2010; Jun et al., 2014). It is therefore possible that distinct populations of DRN neurons innervate different sensory regions of the brain in a modality-specific manner. This agrees with reports on the high degree of heterogeneity among the DRN serotonergic neurons and the existence of “non-overlapping” populations of these neurons that innervate distinct areas of the brain (Abrams et al., 2004; Hale and Lowry, 2011; Gaspar and Lillesaar, 2012; Fernandez et al., 2016).

It is known that superficial pyramidal cells in the ELL-LS respond poorly to beat frequencies smaller than 20 Hz and best to one particular class of chirps (small chirps) when they occur on a background of these frequencies (Marsat et al., 2009; Marsat and Maler, 2010). This property is due to the cancellation of redundant beat signals at low frequencies through an indirect feedback pathway, which is most efficient at a |Df| <20 Hz (Bol et al., 2011). Moreover, small chirps that occur on low beat frequencies synchronize primary afferent activity, whereas chirps occurring on higher beat frequencies desynchronize them (Benda et al., 2006; Walz et al., 2014). Information on the occurrence of small chirps on low beat frequencies is therefore conveyed to subsequent stages of sensory processing and eventually motor centers in a distinct neural code. Beats with such small |Df| values occur during interaction with same-sex conspecifics of similar size (Zakon and Dunlap 1999), and thus in a context of maximum rivalry. Interestingly, we found that for stimuli with small |Df| values there was a negative correlation between the number of chirps produced by the fish and the 5-HT current AUC (Fig. 7*D*). This correlation, however, was weak: variance in the AUC explained only 12% of the variance observed in the number of chirps produced at low |Df| values. Moreover, the correlation was absent for stimuli with large |Df| values. We expect this correlation to be tighter at the level of the motor nuclei that produce chirps [central posterior/prepacemaker nucleus (CP/PPn)], which also receive extensive, sexually dimorphic 5-HT innervation (Telgkamp et al., 2007). Dual FSCV recordings in CP/PPn and ELL-LS in response to conspecific signals could further clarify the relationship between 5-HT transients in these regions and behavioral output. The number of chirps produced is additionally influenced by memory and habituation (Harvey-Girard et al., 2010), and is under the control of telencephalon, which itself receives dense serotonergic innervation (Johnston et al., 1990). Further studies are required to characterize sensory modulation of serotonin release at the level of these and other neural circuits that are involved in the processing and production of communication signals, and to discover the relationship between those dynamics and the number of chirps produced.

We found that in trials in which the fish produced chirps, there was a small but significant positive correlation between the 5-HT response AUC and |Df| (Fig. 7*C*). Walz et al. (2014) proposed that small chirps could be used to discriminate different behavioral contexts, as determined by the beat frequency. It is therefore possible that, when a fish is actively engaged with a conspecific and produces chirps, it can better discriminate the behavioral context (i.e., the sex and body size of the conspecific) and fine-tune its 5-HT response on-line accordingly.

It has been shown that changes in the baseline levels of 5-HT depend on the types of behaviors the fish are engaged in (Zubizarreta et al., 2012; Silva et al., 2013). Our experiments additionally show that the behavioral actions of an animal could affect the second-by-second 5-HT dynamics in a context-dependent (i.e., Df-dependent) manner. When we pooled all chirp trials and compared their 5-HT AUCs with those of no-chirp trials, we found that for small |Df| values the AUC was significantly smaller in the trials in which the fish produced chirps. Therefore, although the correlation between the number of chirps and 5-HT AUC was weak on a trial-by-trial basis, on average the amount of 5-HT response was significantly larger in the trials in which the fish did not chirp. This is in line with previous results showing that, on average, the injection of 5-HT and the stimulation of raphe reduces chirping in response to stimuli with low |Df| values (Maler and Ellis, 1987; Deemyad et al., 2013). Additionally, Deemyad et al. (2013) reported that 5-HT injection renders ELL-LS pyramidal cells more sensitive to chirps. Here we report that for small |Df| values when the fish produces chirps, 5-HT levels are lower and, therefore, based on the results of Deemyad et al. (2013), there should be a decrease in the sensitivity of LS pyramidal cells to fish's own chirps as well as those emitted by a conspecific. On the other hand, in trials in which the fish does not chirp, that is, when the fish is just “listening,” more 5-HT is released, and therefore the ELL-LS cells should be more sensitive to chirps. Interestingly, the 5-HT response was not significantly different between chirp and no-chirp trials in the first 5 s of stimulation (Fig. 8*C*), indicating that the chirping of the fish might be linked to a reduction in 5-HT release. Further experiments are required to prove the causality between chirping and the reduction of 5-HT release for small |Df| stimuli.

The role of 5-HT in the processing of stimuli with large |Df| values is yet to be understood. Deemyad et al. (2013) reported that 5-HT increases the sensitivity of ELL-LS pyramidal neurons to beats at low but not high |Df| values. Nevertheless, we found that stimuli with large |Df| values were as efficient in triggering endogenous 5-HT release as those at small |Df| values, as there was no significant difference in the 5-HT AUC for small versus large |Df| values. Interestingly, however, unlike responses to small |Df| values, the responses to large |Df| values were not affected by the chirping of the fish (Fig. 8*B*,*C*). This could result in high sensitivity to the chirping of a conspecific at high |Df| values, regardless of the chirping behavior of the fish.

Therefore, in the context of maximal rivalry (i.e. for beat frequencies with small |Df| values), the fish is less sensitive to the chirping of another fish when the fish itself is chirping, and is more sensitive when the fish is “listening” (i.e. not chirping). On the other hand, in the context of interaction with opposite-sex conspecifics, or same-sex conspecifics of different size, the fish is listening to the chirping of the other fish, whether or not he is chirping himself. To corroborate these findings, it will be very interesting to record and compare the responses of LS pyramidal neurons to chirps between the trials in which the fish produces chirps and those in which it does not.

5-HT receptors have not yet been cloned in weakly electric fish, and their anatomical distribution is currently unknown. However, *in vitro* pharmacological experiments have suggested the presence of 5HT_2_-like receptors in the ELL-LS (Larson et al., 2014). Application of the 5HT_2_ receptor blocker ketanserin occludes the effect of 5-HT on the bursts produced by ELL-LS pyramidal cells. We cannot be certain which subtypes of 5-HT receptors were activated in our experiments based on the aforementioned pharmacological experiments, because the doses of 5-HT used in those experiments (millimolar range) were orders of magnitude larger than those we found for endogenous 5-HT release (nanomolar range). No direct information is available on the receptor subtypes expressed at the level of the motor output nuclei that generate chirps (CP/PPn) either. However, systemic injection of the 5HT_2_ receptor agonist 2,5-Dimethoxy-4-iodoamphetamine hydrochloride (DOI) DOI was reported to increase chirping, whereas the injection of agonists and antagonists of 5HT_1B/1D_ had no effect on chirping (Smith and Combs, 2008). The same study showed that the injection of the 5HT_1A_ receptor agonist 8-Hydroxy-2-(dipropylamino)tetralin (8-OH-DPAT) specifically increased the production of high-frequency chirps. These chirps are thought to be emitted in the context of courtship and were not observed in our experiments. The cloning of various 5-HT receptors and further anatomical studies are required for unraveling the distributions of the various 5-HT receptor subtypes in different areas of the brain. Our results provide, however, critical information about physiologically relevant doses of 5-HT that can be used for future pharmacological experiments.

Finally, we propose that the variability in 5-HT release dynamics can at least partially account for the heterogeneities that are observed in the responses of the LS neurons (Ávila-Åkerberg et al., 2010; Marsat and Maler, 2010), and possibly sensory neurons of other modalities that receive dense 5-HT innervation. Marsat and Maler (2010) have shown that such heterogeneity improves the discrimination of a second class of chirps (large chirps) that are emitted mostly during interactions with the opposite sex (i.e., those associated with large |Df| values). 5-HT release in LS may thus help the fish discriminate the detailed time-course of large chirps by introducing heterogeneity into the response of LS neurons.

There are many parallels between our findings in the electrosensory system and the auditory system of mammals. For example, HPLC studies have shown that auditory white noise increases 5-HT levels in the dorsal cochlear nucleus (DCN) in the rat (Cransac et al., 1998). 5-HT in turn increases the excitability of DCN principal cells in part via 5-HT_2A/2C_ receptors that are similarly implicated in the regulation of excitability of LS pyramidal cells (Larson et al., 2014; Tang and Trussell, 2015). In the IC of bats, 5-HT enhances the signal-to-noise ratio of the response to communication calls (Hurley and Pollak, 2005). Although an earlier HPLC study did not show an increase in 5-HT level in the IC of rats in response to auditory white noise stimuli (Cransac et al., 1998), voltammetric recordings of 5-HT level in response to such stimuli have shown a clear transient increase in 5-HT level that declines after stimulus offset (Hall et al., 2011). The modality specificity of the 5-HT response has been reported in the IC of mice where auditory, but not visual or olfactory, stimuli evoke elevations in 5-HT levels (Hall et al., 2010). The effect of past experience on 5-HT dynamics has been demonstrated in IC of mice, where interaction with an intruder results in an increase in the 5-HT level in the IC of the resident mouse, and this effect is augmented upon a second bout of interaction with the intruder (Hall et al., 2011). DRN in mice receives monosynaptic inputs from various sensory areas, including a prominent input from IC in the midbrain (Ogawa et al., 2014; Pollak Dorocic et al., 2014), which is thought to be analogous to TS of weakly electric fish (Carr et al., 1981).

During interaction with conspecifics, weakly electric fish sometimes engage in “echo response,” where the fish chirp at each other back and forth with interchirp latency ranging between 200 and 600 ms (Hupé and Lewis, 2008). The processing of conspecific signals and behavioral responses therefore occurs at a subsecond timescale in weakly electric fish, and likely in other model systems. Our findings shed light, for the first time, on the subsecond dynamics of 5-HT level changes in a sensory brain area resulting from sensory stimulation *in vivo,* and suggest that an behavior of an animal can modulate these dynamics on a second-by-second basis.

## Synthesis

The decision was a result of the Reviewing Editor Carmen Sandi and the peer reviewers coming together and discussing their recommendations until a consensus was reached. A fact-based synthesis statement explaining their decision and outlining what is needed to prepare a revision is listed below. The following reviewers agreed to reveal their identity: Stephane Marinesco, Jan Benda

Although much is known about the effects of modulators, such as serotonin, in the nervous system, very little is known about how, where, and at what time scales serotonin is released in the context of sensory (communication) stimuli. This manuscript -well written and illustrated- elegantly addressed these important questions and provides interesting insights. This is particularly relevant as, while there is much focus on the function of serotonin at a molecular level in the literature, studies that quantify serotonin release in an in vivo system in the context of natural-like stimuli are largely missing. The manuscript fills this gap with sub-second resolution measurements. The manuscript is mostly very well written and illustrated.

Specifically, this manuscript studies 5-HT release in the electrosensory organ of the weakly electric fish Apteronus Leptorhynchus using an elegant fast-scan cyclic voltammetry technique. These fish constantly generate an electric field with a characteristic frequency and can detect that of conspecifics. The presence of a foreign electric field with a frequency close to that of the animal can be the trigger of behavioral responses including a change in the frequency of the emitted signal intended to minimize jamming, or the emission of a short communication signal called chirp. Pharmacological studies indicate that chirp emission is under the inhibitory control of the neuromodulator 5-HT. However, the actual release of 5-HT in the electrosensory organ in response to conspecific signals had never been studied.

The authors used fast-scan cyclic voltammetry to detect 5-HT release evoked by an electric field resembling a conspecific signal. They found a 5-HT signal in a subregion of the electrosensory organ that specifically receives 5-HT innervation, whereas 5-HT was undetectable in other regions devoid of 5-HT fibers. In addition, there was strong resemblance between electrochemical signals detected in vivo and the shape of standard voltammograms recorded in vitro. Finally, 5-HT signal increased in response to citalopram, a 5-HT reuptake inhibitor. Overall, the demonstration of the serotonergic identity of the signal is convincing.

Subsequently, the paper analyzed the relationship between the electrochemical 5-HT signals recorded during the presentation of a conspecific signal and the emission of chirps. This is the most interesting part of the paper, but unfortunately, the weakest. The authors conclude that 5-HT release is smaller in response to external fields that produced chirps compared to those that did not produce chirps, and that chirp production results in changes in 5-HT release. However, the pharmacological data from the literature would rather suggest the reciprocal logical relationship, i.e. smaller 5-HT release would disinhibit chirp production.

The authors should clearly address the following issues in their revision:

- What are the experimental facts that allow them to conclude on the logical link between 5-HT release and chirp emission?

- Since they have access to high temporal resolution recording, they could compare the latency of chirp emission and 5-HT release. What comes first? What is the mean latency of chirp emission when detecting an external field?

- The authors did not analyze failure rates. This information is usually useful to understand a model system.

- It is also very important to understand how reliable are the 5-HT release events and the chirps. What is the failure/success rate of 5-HT release (i.e. the probability that an external field produces 5-HT release or not). Does this vary with Df? Are there any similarities with success/failure rates of chirp emissions?

- In the discussion part, a better explanation of the different 5-HT receptor subtypes that are possibly involved in the modulation of chirp production should be provided. This is especially relevant when discussing the time-course of the 5-HT signal. For example, 5-HT detected here is necessarily extrasynaptic, and it is possible that 5-HT is first released at synapses, accumulates and then diffuses to extrasynaptic sites with a couple seconds delay. Are the 5-HT receptors subtypes involved in the modulation synaptic or extrasynaptic?

- In addition, it would be important to discuss the difference between exogenous 5-HT application (by a microinjection for example) or synaptically released 5-HT. Again, is there any evidence that microinjections and physiological 5-HT release could activate different receptors?

- From the novelty point of view, the statements made indicating that this study is first of its kind is not correct and they should be eliminated, in addition to referencing the previous literature. Specifically, studies by Hall and Hurley have already performed 5-HT recordings in vivo in the mouse superior colliculus, although their recordings were on a minute time-scale (Hall et al. 2011, Hall et al. 2011). In addition, 5-HT detection at a sub-second time resolution using carbon fiber microelectrodes have already been performed in the Aplysia nervous system in the context of sensory modulation (Marinesco and Carew 2002, 2006), although these were in vitro studies. Overall, it is factually true that the measurements described here are the first in vivo and subsecond 5-HT recordings, but they represent an incremental improvement over the current state of the art, and therefore such claims about novelty are not deserved.

In addition, the authors should also address the following specific issues:

- page 6 lines 140-142 (normalization of the citalopramresponses) and Fig 2E: I am not entirely sure whether I understood your normalization procedure right. The problem might just be that you do not have a properly labeled y axis on that plot. The blue curve in Fig 2E should have its peak at about 1, right? Can you please add a y axis to this plot, labeled 'normalized current' with a zero and a one on it?

- page 7 line 164: Dfranges: please list the DFs you actually used.

- page 7 line 178 (population analysis on normalized currents): this I do not understand, in particular why the current values in the corresponding figures ar so much smaller than one. For each fisch, did you take the single maximum current that you recorded for normalization? If yes, is this really a good idea to base the normalization on a single value that so easily can be distorted by some noise? Why don't you normalize it like in the citalopram experiment to the average peak current? Here you would take the peak currents of the responses to some DF. If no, you need to better explain what you actually did.

- page 7 bottom, area under the curve. This is the integral under the curve like in Fig 5B from 0 to 20s, right? The differences you get for the AUCs in the subsequent analysis appear rather small compared to the curves of the peak current you show. I guess one reason for that is that in the first approx 5 seconds the peak current is still small and has not reached its steady state level. I would suggest to take the integral only over the last 10 seconds of the response. Is the other reason for the rather small difference in AUCs the huge variability of the response amplitudes?

- page 8 line 205: "a current in MS with the same cyclic voltammogram" better say "a similar".

- page 9 line 231: you could emphasize the fact that not each trial evoked a serotonin response.

- page 10 center paragraph: add units to the AUC mean and standard deviation. Consider to restrict the AUC to the last 10 sec of the stimulus (see above).

- page 10 line 255 and page 12 line 293: "For most Esense-stim trials, the 5HT reponse..." please state what "most" means, i.e. what fraction of trials had a response below 2 sec.

- page 11 line 272 (no significant correlation between Df and the AUC): is this trua also for individual fish?

- page 11 para starting at line 273: what about a plot showing AUC versus Df separated for chirping and non chirping fish? Would you get correlations?

Figures general comments:

unify how you label axis (Figure one drops out): Capital name + space + unit in brackets.

unify whether you have a space between numbers and unit or not (e.g. fig 2 A stimulus description, fig 2B VC tick labels)

in fig 6 A you state the number of fish in the plot. Why not doing this everywhere? Or nowhere?

Figure 1:

Was the cyclic voltammogram presented at the bottom obtained in a standard 5-HT solution?

bottom panels: Volt is V not v!

current has a unit!

make the VC (bottom right) similar to the ones shown in the other figures, i.e. have the y-axis going through 0.2 volt. Indicate on the x-axis -0.1, 0.0, 0.2 and 1 V.

Figure 2:

A: stimulus description: add space after each comma

B, D: add an arrow from the VC to the corresponding time in the peak current plot (as in Fig 5B).

B, D, E: add a proper time axis to the plots, so that they are better comparable to the plots in A and B, make them the same length (except maybe E). A proper y-axis would also help, in particular in E (see comment above).

A, C: horizontal dashed line marks the peak current? vertical dashed line the time point of the VCs? Please mention this in the caption.

Figure 3 C: spontaneous serotonin releases: do you know whether the fish produced spontaneous chirps and if yes whether they are related to the spontaneous serotonin releases?

Figure 4: add vertical dashed lines in A and arrow in B to indicate position of VC?

Figure 5: I would like to see some individual blank responses, i.e. the trials that make up the black average in B. Likewise instead of or in addition to A, would it mak e sense to show the individual curves that make up the the red average in B, color coded by df? Or show the averages of the different Dfs.

Figure 5 caption B: "Time course of ... current evoked by ... and spontaneosly in the same group" a verb is missing after "spntaneously"

Fig 7 A: how many fish?

How does this look like for a single (good chirping) fish?

Statistics:

The authors analyze 5-HT release evoked by external electric fields characterized by their frequency difference (Df) with the animal's own field. They found that 5-HT release did not depend on Df, although chirps were emitted exclusively by low Df external fields. For external fields with low Df, they found that 5-HT signals evoked when the animal chirped were significantly smaller than 5-HT release evoked by fields with high Df. This difference was not present in 5-HT signals evoked by external fields that did not produce chirps. This analysis, however, appears biased. The comparison that one would expect would be between 5-HT release evoked by low Df signals that evoked or did not evoked chirps. Is this comparison significant? If not, the conclusion drawn by authors appears quite weak. Here, the authors compared 5-HT signals that are evoked by signals of different Df, which adds another variable. Such comparison in which multiple factors change is not recommended.

Ethics:

The methodological procedures and ethical-sensitive details should be explained in much greater detail. Here, the authors refer to a preceding paper that itself, refers to another study. Please provide details on the steps that were taken to minimize suffering. Was pain appropriately controlled and habituation done here? Please, expand in the methods section to make sure that all ethically-relevant issues where appropriately controlled.
